# DNA primase subunit 1 deteriorated progression of hepatocellular carcinoma by activating AKT/mTOR signaling and UBE2C-mediated P53 ubiquitination

**DOI:** 10.1186/s13578-021-00555-y

**Published:** 2021-02-23

**Authors:** Mengqi Zhu, Mengna Wu, Saiyan Bian, Qianqian Song, Mingbing Xiao, Hua Huang, Li You, Jianping Zhang, Jie Zhang, Chun Cheng, Wenkai Ni, Wenjie Zheng

**Affiliations:** 1 Research Center of Clinical Medicine, Affiliated Hospital of Nantong University, 20 Xisi Road, Nantong, 226001 Jiangsu China; 2 School of Medicine, Nantong University, 19 Qixiu Road, Nantong, 226001 Jiangsu China; 3 Department of Oncology, Affiliated Hospital of Nantong University, 20 Xisi Road, Nantong, 226001 Jiangsu China; 4 Department of Radiology, Wake Forest School of Medicine, One Medical Center Boulevard, Winston-Salem, 27157 NC USA

**Keywords:** DNA primase subunit 1, Hepatocellular carcinoma, P53, AKT/mTOR, Molecular target

## Abstract

**Background:**

DNA primase subunit 1 (PRIM1) has been reported as a novel oncogene in several cancer types. However, its roles in hepatocellular carcinoma (HCC) remain unclear. This study aimed to investigate underlying mechanisms of PRIM1 and identify it as a potential molecular target for HCC.

**Methods:**

Hub genes were screened between HCC tissues and normal liver tissues in 3 gene expression omnibus (GEO) datasets and the cancer genome atlas (TCGA). The expression features and prognostic value of one of the hub genes PRIM1 were analyzed by bioinformatic analyses and immunohistochemistry. Loss-of-function and gain-of-function studies were used to investigate the regulatory role of PRIM1 in HCC cells. Real-time (RT)-qPCR, western blotting, and ubiquitin immunoprecipitation assays were performed to explore the underlying mechanisms. The xenograft model was employed to detect the roles of PRIM1 in tumor growth in vivo. Finally, the 3D spheroid model was conducted to validate the role of PRIM1 in tumor growth and sorafenib resistance.

**Results:**

The hub genes of HCC were screened in multiple bioinformatic datasets. PRIM1, as one of the hub genes, was significantly overexpressed in HCC tissues in mRNA and protein levels. In addition, high expression of PRIM1 indicated poor prognosis of HCC patients in TCGA, ICGC, and Nantong cohorts. Overexpression of PRIM1 promoted the proliferation, migration/invasion, and sorafenib resistance of HCC cells, with the decrease in apoptosis and cell cycle arrest. Mechanically, PRIM1 facilitated epithelial-mesenchymal transition (EMT) process and the activity of PI3K/AKT/mTOR signaling of HCC cells. Additionally, PRIM1 could cause the ubiquitination and degradation of P53 by upregulating Ubiquitin Conjugating Enzyme E2 C (UBE2C). Furthermore, knockdown of PRIM1 significantly inhibited the growth of xenograft tumors and HCC cells-derived spheroids with enhanced sorafenib resistance.

**Conclusion:**

This study implies that PRIM1 may play a key role in the progression of HCC and may serve as a potential target for HCC treatment.

## Introduction

Hepatocellular carcinoma (HCC) is one of the leading causes of cancer-related mortality, with ~ 1 million new cases worldwide. Risk factors of HCC include hepatitis B virus (HBV) or hepatitis C virus (HCV) infections, aflatoxin B1 exposure, alcohol abuse, and metabolic syndrome [1]. Surgery is optimal for the HCC patients at early stage. However, 50% of HCC patients were diagnosed at advanced stages, for whom ablation, transarterial chemoembolization are optional strategies [2]. Furthermore, systematic therapy such as multiple-target tyrosine kinase inhibitor sorafenib have shown efficacy in improving the outcomes of HCC patients at late stages. Regorafenib, cabozantinib, and ramucirumab were approved as second-line treatments for advanced HCC [3]. Despite of the remarkable progression in surveillance, diagnosis and management, the overall outcome of HCC patients remains poor due to increasing recurrence and chemoresistance [4]. Therefore, it is warranted to better investigate the biological and molecular processes in HCC and provide novel targets for HCC.

DNA primases synthesize primers for DNA polymerases-mediated DNA replication. DNA primases are also defined as DNA-dependent RNA polymerases. The polymerases complex includes the catalytic subunit POLA1, the regulatory subunit POLA2, as well as the primase complex subunits DNA Primase Subunit 1(PRIM1, 49 kDa) and DNA Primase Subunit 2 (PRIM2, 58 kDa) [5]. PRIM1 mapping at chromosome 12q13 is the smallest subunit within a polymerase α (Pol α) heterotetrameric complex, which plays a crucial role in DNA replication by facilitating catalytic function and synthesizing RNA primers as starting point for the replication of the leading and lagging strand, while PRIM2 had no similar enzymatic activities [6]. Baranovskiy AG et al. manifested the precise crystal structure and overall organization of PRIM1, suggesting the fundamental roles of dramatic conformational changes in its interaction with DNA and RNA, as well as the elongation of RNA synthesis [7]. Given its importance function in DNA replication, PRIM1 deficiency has been correlated with replication-associated disorders. Parry DA et al. recently described a distinct form of microcephalic dwarfism associated with PRIM1 mutations [8]. Moreover, PRIM1 deficiency was also correlated with primary ovarian insufficiency [9] and type 2 diabetes mellitus [10]. In addition, missense mutation of PRIM1 may cause extensive apoptosis of retinal neurons through activation of the DNA damage checkpoint and tumor suppressor P53, with no alterations in cell proliferation [11]. For its potential roles in oncogenesis, amplifications of PRIM1 were firstly found in osteosarcoma [12]. Recently, emerging evidence has shown that PRIM1 acts as an oncogene that enhances the aggressive behaviors of tumor cells. Lee WH et al. manifested that depletion of PRIM1 inhibited the cell cycle progression and induced DNA damage of the breast cancer cells through inactivating G2/M cell cycle checkpoint [13]. Job et al. demonstrated that PRIM1 inactivation sensitized colorectal cancer cells to ATR and CHK1 inhibitors, suggesting PRIM1 or other primase subunits could be novel targets for individualized tumor therapeutic approaches [14]. Besides, PRIM1 was also identified as a predictor that indicated poor survival of tumor patients. Though recent studies described the abnormal expression features of PRIM1 [15], the mechanisms underlying its oncogenic roles have not been elucidated.

In the current study, we screened hub genes of HCC through integrated bioinformatic analyses. PRIM1 was further selected to comprehensively analyze its expression profiling and clinical values. In addition, we also investigated the roles of PRIM1 in phenotypic manifestations and the underlying molecular mechanisms. This study may provide new insights into the HCC progression and therapeutic targets for HCC treatment.

## Materials and methods

### Clinical samples

Tissue samples of Nantong cohort were collected from 135 HCC patients who underwent surgery between January 2012 and October 2014 at Affiliated Hospital of Nantong University (Nantong, Jiangsu, China). The study was performed in compliance with the Helsinki Declaration and approved by the ethic committee of Affiliated Hospital of Nantong University (TDFY2018-025).

### Cell culture and reagents

HepG2 (catalog number, #SCSP-510), Hep3B (catalog number, #SCSP-5045), and BEL-7404 (catalog number, #TCHu64) were purchased from the Cell Bank of the Chinese Academy of Sciences (Shanghai, China). MHCC97H (catalog number, #ZQ0020), MHCC97L (catalog number, #ZQ0019), and HCCLM3 (catalog number, #ZQ0023) were purchased from Zhong Qiao Xin Zhou Biotechnology (Shanghai, China). Cells were maintained in Dulbecco’s modified Eagle’s medium (Gibco, USA) supplemented with 10% fetal bovine serum (FBS; Gibco, USA) and 1% penicillin/streptomycin solution.

### Data preprocessing

Microarray data of GSE25097, GSE121248, GSE1112790 was downloaded from the National Center for Biotechnology Information (NCBI) Gene Expression Omnibus (GEO, http://www.ncbi.nlm.nih.gov/geo/) database. Dataset GSE25097 contains 37,582 probe IDs across 512 samples, including 243 non-tumor and 268 tumor samples from Hepatocellular carcinoma (HCC). GSE121248 has 54,675 probe IDs across 107 samples from HCC, including 37 non-tumor and 70 tumor samples. The total expression matrix of GSE25097 has 54,613 probe IDs across 198 samples, including 15 non-tumor and 183 tumor samples. For multiple probes corresponding to one gene, their average expression value was taken as the gene expression value. After that, gene expression values were normalized using preprocessCore package (version 1.28.0, http://www.bioconductor.org/packages/release/bioc/html/preprocessCore.html), and were performed with log2 transformation. Level 3 gene expression matrices of 415 HCC patients were retrieved from TCGA data portal (The Cancer Genome Atlas Liver Hepatocellular Carcinoma (TCGA-LIHC) data; https://cancergenome.nih.gov/). Out of 415 samples, 365 were solid tumor tissue samples (HCC) and 50 were the adjacent non-tumor tissue samples (controls). The clinical features of HCC patients in TCGA and International Cancer Genome Consortium (ICGC) are presented in Table 1.

**Table 1:** The clinical characteristic information of the HCC patients in TCGA and ICGC

Characteristics	TCGA (%)	ICGC (%)
Age		
< 60	177 (*47.32*)	50 (*27.59*)
≥ 60	196 (*52.41*)	210 (*72.41*)
NA	1 (*0.27*)	
Gender		
Male	253 (*67.65*)	192 (*73.71*)
Female	121 (*32.35*)	68 (*26.29*)
Survival status		
Alive	238 (*63.64*)	214 (*81.47*)
Dead	130 (*34.76*)	46 (*18.53*)
NA	6 (*1.60*)	
Stage		
I	173 (*46.26*)	40 (*15.52*)
II	87 (*23.26*)	117 (*45.69*)
III	85 (*22.73*)	80 (*30.60*)
IV	5 (*1.34*)	23 (*8.19*)
NA	24 (*6.42*)	
Histological grade		
G1	55 (*14.71*)	NA
G2	178 (*47.59*)	NA
G3	124 (*33.16*)	NA
G4	12 (*3.21*)	NA
NA	5 (*1.34*)	
T classification		
T1	183 (*48.93*)	NA
T2	95 (*25.40*)	NA
T3	80 (*21.39*)	NA
T4	13 (*3.48*)	NA
NA	3 (*0.80*)	
N classification		
N0	254 (*67.91*)	NA
N1	4 (*1.07*)	NA
NX	115 (*30.75*)	NA
NA	1 (*0.27*)	
M classification		
M0	268 (*71.66*)	NA
M1	4 (*1.07*)	NA
MX	102 (*27.27*)	

*NA* not available

### Differential expression analysis

Linear models for microarray data (Limma; http://www.bioconductor.org/packages/release/bioc/html/limma.html) was the R package that analyzed gene expression microarray data, especially the use of linear models for the assessment of differential expression. DESeq2 (version 1.18.1) package in R was used for differential expression analysis of the sequencing data. Significantly differentially expressed genes were accepted as |log2FC|> 1 and adjusted P-value < 0.01.

### Functional analysis

*Pathway database.* Reactome (http://www.reactome.org) is a manually curated open-data resource of human pathways and reactions, an archive of biological processes and a tool for discovering potential functions. Gene sets derived from the Reactome pathway database were downloaded from the MSigDB Collections. *Enrichment test.* Functional enrichment based on the Reactome and GO terms databases was assessed by hypergeometric test, which was used to identify a priori-defined gene sets that showed statistically significant differences between two given clusters. The test was performed by the clusterProfiler package. We further corrected the test P-values by the Benjamini–Hochberg and less than 0.05 was considered as statistically significant. The GO enrichment was visualized using the GOplot package.

### Survival analysis

The Kaplan–Meier analyses were performed in TCGA-LIHC and ICGC samples. Kaplan–Meier (KM) curve with a log rank test was used for presenting the results of survival analysis. The ‘survival’ R package (http://cran.r-project.org/web/packages/survival/index.html) was subjected to survival analysis.

### Gene set enrichment analysis (GSEA)

GSEA 3.0 software (http://www.broad.mit.edu/gsea) was performed to predict the potential functions and mechanisms mediated by PRIM1 in TCGA LIHC and ICGC databases. The reference was obtained from Molecular Signatures Database (MSigDB, http://software.broadinstitute.org/gsea/msigdb). HCC cohort was stratified into high and low PRIM1 groups based on the median PRIM1 expression. Thresholds were determined after 1000 permutations. Pathway enrichment was determined in a weighted manner. P value/FDR and (normalized enrichment score) NES were used to screen potentially enriched terms. The assay was conducted by the clusterProfiler package.

### Multiple online bioinformatic databases

UALCAN database (http://ualcan.path.uab.edu/) was used to evaluate PRIM1 expression in HCC samples in various subgroups. The LinkedOmics database (http://www.linkedomics.org/ login.php) was used to predict the functions and pathways modulated by PRIM1 by conducting over-representation enrichment analysis (ORA). The correlation of PRIM1 with TP53 and UBE2C was analyzed by TIMER database (https://cistrome.shinyapps.io/timer/). Kaplan–Meier analyses of HCC patients in TCGA dataset were performed by Kaplan–Meier Plotter (http://kmplot.com/).

### Plasmid transfection

Lv-PGK > EGFP/T2A/Puro lentivirus plasmids for PRIM1 knockdown were constructed by Cyagen (Shanghai, China). The sequence of the Kd-PRIM1 was GATTGATATAGGCGCAGTATACTCGAGTATACTGCGCCTATATCAATC. Lentivirus vectors were infected into MHCC97H cells with a multiplicity of infection ranging from 10 to 20. Then, stalely transfected cells were screened by using puromycin (5 μg/mL, Sigma-Aldrich). The sequence of the Kd-UBE2C was CCGGCCTGCAAGAAACCTACTCAAACTCGAGTTTGAGTAGGTTTCTTGCAGGTTTTTG. Kd-UBE2C/PTSB-SH-copGFP-2A-PURO and OE-UBE2C/pTSB02-GFP-PURO plasmids were obtained from Transheep (Shanghai, China). The plasmids pEX4 EGFP/T2A/Kan/Neo for overexpression of PRIM1 were constructed by GenePharma (Shanghai, China).

### Cell counting kit-8 and colony formation assays

Cell growth was evaluated by Cell Counting Kit-8 assay (CCK-8; Dojindo Laboratories, Kumamoto, Japan) according to the manufacturer’s protocols. In Brief, HCC cells were cultured in 96-well plates at a density of 1 × 10^3^ cells well/100  μL. After 12 h, 10  μL of CCK-8 solution was administrated into each well at indicated time points (24 h, 48 h, and 72 h). Following incubated at 37 °C for 2 h, the plates were measured at the wavelength of 450 nm. The assay was independently repeated for three times. For colony formation assays, HCC cells of each group were seeded in the six-well plate at a density of 500/well. After incubation for 14 days, the samples were fixed in 4% paraformaldehyde (PFA) for 30 min and stained with 0.1% crystal violet solution.

### Transwell assays

The invasion and migration assay were performed by using the 8-μm Transwell chambers (Corning, Acton, MA, USA). For the invasion assay, 200 μL of MHCC97H or HepG2 cells of each group were plated in the upper chambers pre-coated with Matrigel (BD, CA, USA) mixed in DMEM, while the complete medium was plated in the lower chamber. After incubated for 24 h, the upper chambers were fixed in 4% paraformaldehyde and stained in the crystal violet solution. Then, the stained cells were observed under the microscope. For the migration assay, the same protocol was conducted without the administration of the Matrigel. Five fields were randomly selected to calculate the average number of invasion or migration cells.

### Xenograft tumor assay

Four-week-old BALB/c nude mice were obtained from Animal Center of Nantong University (Nantong, China). HCC cells suspended at the density of 5 × 10^6^/100  μL serum-free DMEM were subcutaneously injected into the flanks of the mice. The xenograft tumors were monitored every 3 days for a total of 5 weeks until the sacrifice. The volume was measured as follows: volume = length × width^2^ × 0.5. The procedures of this study were approved by the Animal Care and Use Committee of Nantong University.

### Spheroid model construction

The 3D spheroid model was constructed as previously described [16]. In brief, different groups of MHCC97H cells [PRIM1 Knockdown, PRIM1 Knockdown/UBE2C overexpression, and negative control (NC)] and HepG2 cells (PRIM1 Knockdown, PRIM1 Knockdown/UBE2C overexpression, and NC) were mixed with Collagen I (Meilun Biotechnology, 5 μg/mL) and plated in the Corning^®^ 96 Well Clear Round Bottom Ultra Low Attachment Microplate (Corning, USA) at the density of 2000 cells/200 μL. At the indicated time point of 1st day, 3rd day and 5th day, the formed spheroid were photographed and volumed. Then, on the 5th day, each group was treated with sorafenib (20 μM) and photographed in another 3 days.

### Immunohistochemistry

Human liver and mouse specimens were fixed in formaldehyde and embedded in paraffin. Sections were dewaxed by xylene and dehydrated by serial diluted ethanol. Then the slides were incubated in 0.3% hydrogen peroxide and blocked with 1% BSA solution. Following that, the slides were incubated in anti-PRIM1 (1:200, Abcam, USA), anti-P53 (1:200, Proteintech, USA), anti-UBE2C (1:200, Proteintech, USA), p-AKT (1:200, Proteintech) and p-mTOR (1:200, Proteintech), N-cadherin (1:200, Abcam, USA), E-cadherin (1:200, Abcam, USA), Ki67 (1:200, Proteintech) antibodies at 4 °C overnight. All slides were independently evaluated by two observers. The score of PRIM1 staining was calculated based on the staining intensity and the positive cells. All the percentages/numbers of positive cells were expressed as the average of six randomly selected microscopic fields.

### Immunoprecipitation and protein stability assay

The MHCC97H and HepG2 cell lysates of each group were collected by radioimmunoprecipitation assay buffer (RIPA) for 30 min on ice. Then the lysates were incubated with the primary antibody against P53 (Santa Cruz Biotechnology, USA) overnight at 4 °C. Following that, the samples were incubated with anti-rabbit Ig-IP beads (Rockland Immunochemicals, USA) for 2.5 h at 4 °C. The beads were spun down and rinsed with PBS. Subsequently, the proteins were eluted from the beads by boiling in loading buffer (Bio-Rad) for 5 min and subjected to immunoblotting. For the protein half-life assay, MHCC97H or HepG2 cells in each group were administrated with CHX (100 μg/mL, Sigma, USA) and collected at the indicated time points. IB was used to determine the protein attenuation induced by CHX.

### Flowcytometry

The effects of PRIM1 on apoptosis of HCC cells were analyzed by A FITC-Annexin V Apoptosis Detection Kit (BD Biosciences, USA) according to the manufacturer’s instructions. Cells of each group were pre-suspended in binding buffer and incubated with Annexin V-FITC for 15 min. Following incubation with Propidium iodide (PI), the samples were detected by using a BD FACS Calibur flow cytometer (Becton–Dickinson, USA). For cell-cycle detection, the cells of each group were incubated with PI solution for 15 min and detected by using a BD FACS Calibur flow cytometer. The results were analyzed by Modfit software.

### Reverse transcription quantitative polymerase chain reaction (RT-qPCR)

Total RNA of each group was extracted by TRIzol (Invitrogen, USA) according to the manufacturer’s instructions. cDNA was synthesized by using TaKaRa PrimeScript RT regent kit (TaKaRa, Japan). RT-qPCR was conducted by using SYBR on an ABI 7500 qPCR apparatus (Applied Biosystems, Life Technologies, CA, USA). The glyceraldehyde phosphate dehydrogenase (GAPDH) was used as a loading control. Primer sequences were listed as follows. PRIM1, F, ATGGAGACGTTTGACCCCAC; R, CGTAGTTGAGCCAGCGATAGT. UBE2C, F, GACCTGAGGTATAAGCTCTCGC; R, TTACCCTGGGTGTCCACGTT. GAPDH, F, GGAGCGAGATCCCTCCAAAAT; R, GGCTGTTGTCATACTTCTCATGG.

### Western blotting

Protein of each group extracted by RIPA was separated on a sodium dodecyl sulfate (SDS) gel and transferred onto a polyvinylidene difluoride (PVDF) membrane (Bio-Rad, Hercules, CA, USA). Subsequently, the membranes were blocked in 5% BSA for 2 h and incubated in primary antibodies at 4 °C overnight. Then the samples were further incubated in horseradish peroxidase (HPR) -conjugated secondary antibodies at room temperature for 2 h. The membranes were visualized by using enhanced chemiluminescence (ECL) kit (Millipore, MA, USA). GAPDH (Abcam, Cambridge, UK) was served as an internal control for western blotting.

### Statistical analysis

Data are presented as the mean ± SEM. Statistical analysis was performed using SPSS 19.0 and GraphPad Prism 7.0 (CA, USA). The χ^2^ test and Student’s *t*-test were performed to evaluate the differences between two groups, while one-way or two-way ANOVA was used for multiple group comparisons. Kaplan–Meier curves with log-rank test was performed to evaluate the prognostic value of various variables. All experiments were repeated at least three times. P value < 0.05 was considered statistically significant.

## Results

### Screening hub genes of HCC through integrated bioinformatic analyses

**Fig. 1:**
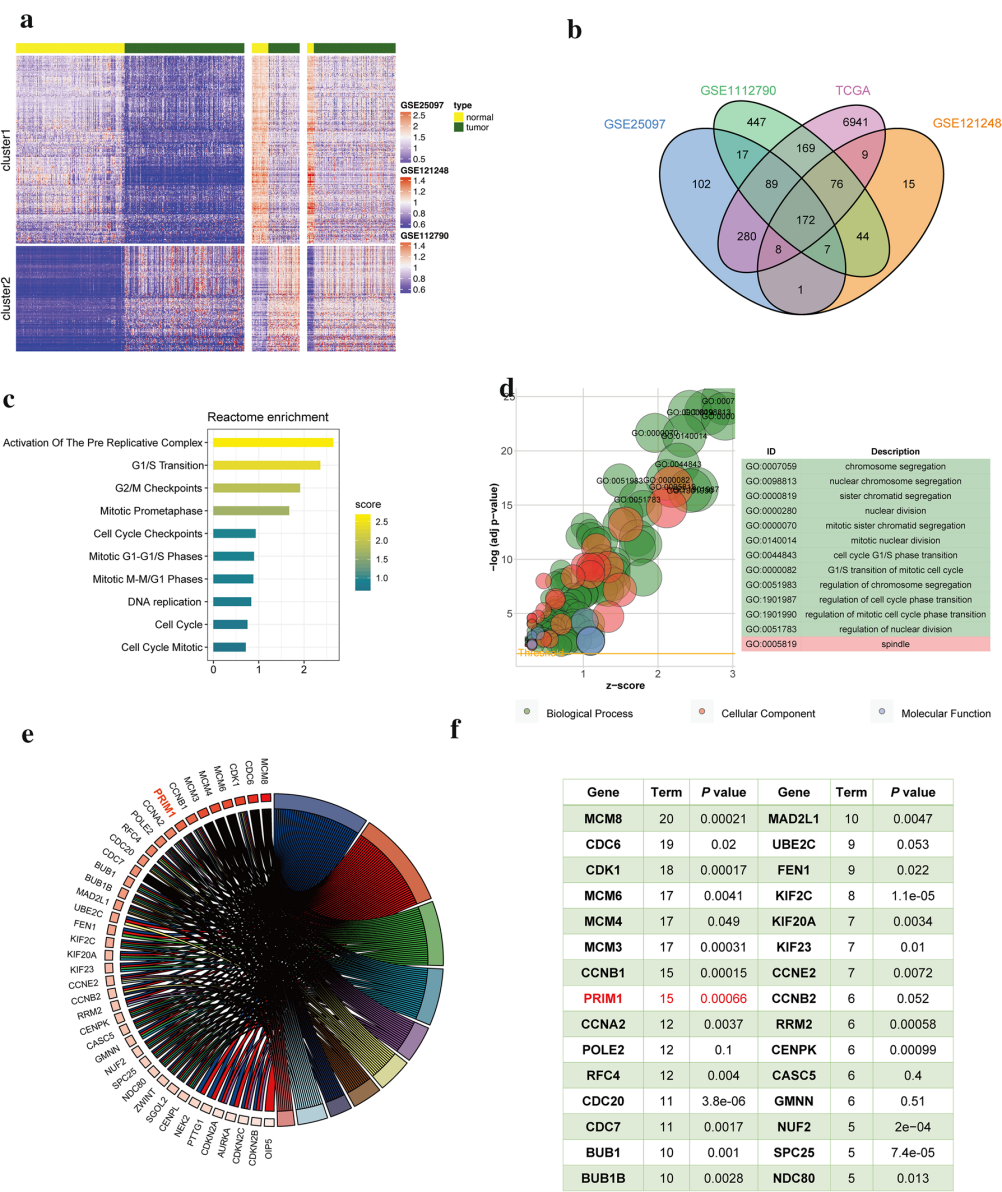
Screening the hub genes of HCC. **a** Heatmap shows the up- and down-regulated genes between tumor and normal samples in different datasets. Genes (row) are clustered to two groups for better visualization and patient samples (column) are ordered according to tumor/normal types. Color scheme is based on z-score distribution, from blue to red Genes (rows) with (log2 fold change) > 1 and adjusted P-value (adj.p) < 0.01 are listed in respective of each dataset. **b** Venn plot of the differentially expressed genes in four datasets. **c** Significantly enriched terms in the Reactome pathway database (y-axis) are shown in bar plots based on the up-regulated genes in the tumor samples compared with normal samples. **d** The bubble plot displays the enrichment of GO terms. The y‑axis indicates the significance of the term (− log10 adjusted P‑value) and the x‑axis indicates the Z‑score. Bubbles indicate the GO terms, with green indicating BP terms, red indicating cellular component terms and blue indicating molecular function terms. The bubble size indicates the gene numbers in the GO terms. **e** Circular plots illustrating the enriched Reactome pathways. The genes are bridged by ribbons to their assigned enriched pathways. The white-to-red color indicates the ranking of the number of enriched Reactome pathways. **f** Log-rank test P-values of the hub genes of HCC

To identify the differentially expressed genes between HCC and normal samples, we performed a comprehensive analysis of the gene expression data from public domain GEO. Three datasets, i.e., GSE25097, GSE121248, and GSE1112790, with both tumor and normal samples were collected from the GEO database. By comparing tumor and normal samples, we identified, respectively 676, 332 and 1021 significantly up-regulated genes in each dataset, which resulted in 501 genes that were consistently overexpressed in three datasets. Hierarchical clustering is performed using the 501 genes across these datasets (Fig. 1a). Besides that, we also downloaded the expression data of HCC patients from TCGA. 7744 genes were shown to be significantly up-regulated in tumor. Finally, we identified 172 up-regulated genes across four datasets, as indicated in the Venn plot (Fig. 1b).

Next, we examined the enriched functions associated with the 172 up-regulated genes in the Reactome pathway database, including activation of the pre-replicative complex, G1/S transition, and G2/M checkpoints (Fig. 1c). To gain more insights into their biological significance, we assessed the enrichment of gene ontology (GO) terms for biological processes (BPs), cellular components (CCs) and molecular functions (MFs). The significant enriched GO terms indicated the cell cycle process with a particular involvement of the G1/S phase transition as well as nuclear division (Fig. 1d). As expected, such annotations indicated the cell cycle processes that were also supported by the Reactome pathway analysis. Further investigation of the enriched Reactome pathways revealed the most frequently involved genes. As shown in Fig. 1e, PRIM1 was one of the top genes that were associated with most of the enriched pathways. Meanwhile, the Log-rank test indicated that PRIM1 might have potentially prognostic significance (P = 0.0006) for HCC patients (Fig. 1f). Then we focused on investigating the potential roles and mechanisms of PRIM1 in HCC.

### Expression and prognostic value of PRIM1 identified in bioinformatic HCC cohorts

**Fig. 2:**
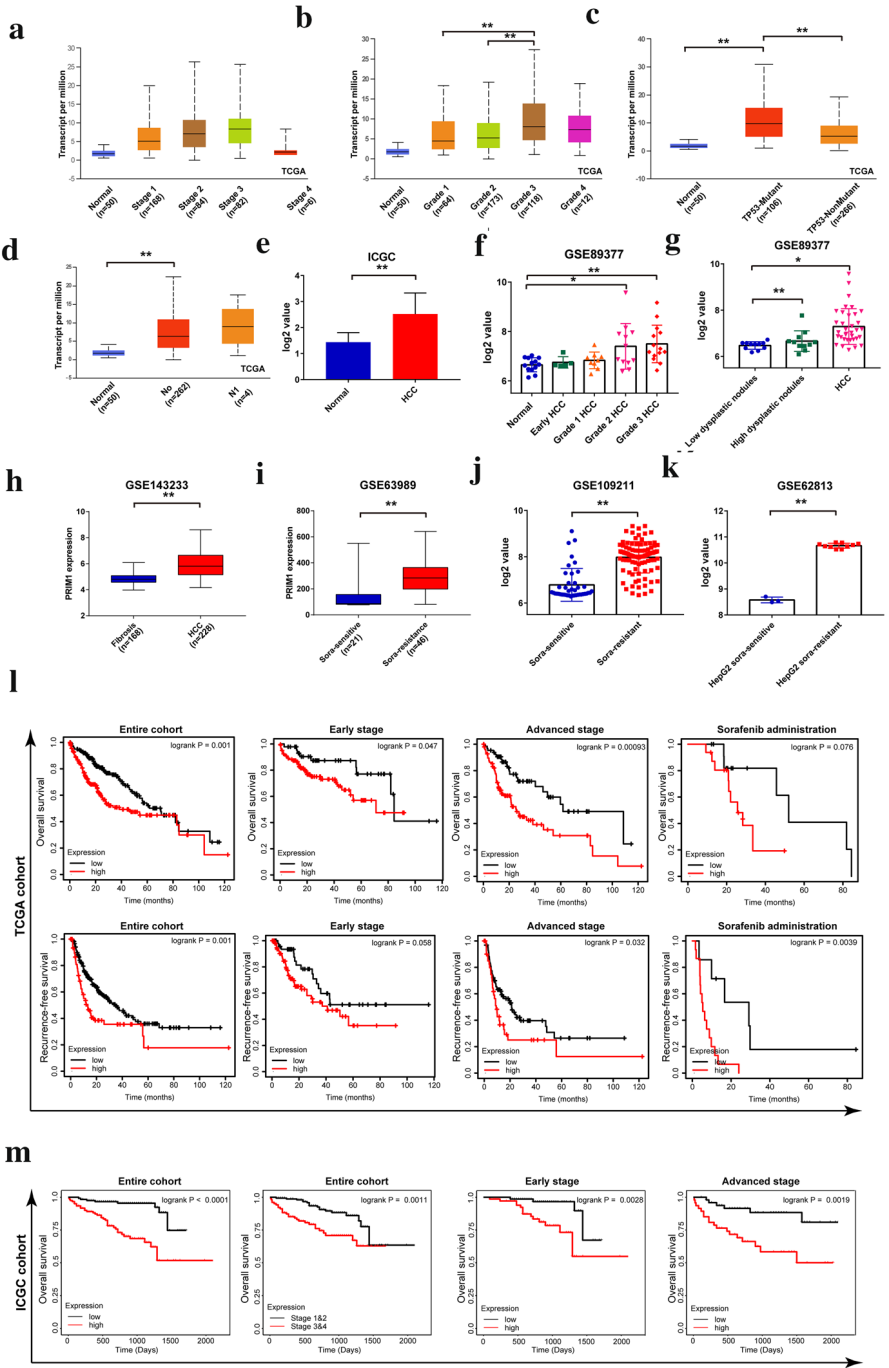
Validating the expression features and prognostic values of PRIM1 in bioinformatic datasets. **a**–**d** The expression levels of PRIM1 in HCC patients with different stages (grades), TP53 mutation, and metastasis status in TCGA LIHC dataset. **e** The expression levels of PRIM1 in HCC tissues and normal liver tissues in ICGC dataset. **f**–**h** The expression levels of PRIM1 in HCC and pre-HCC cases in GSE89377 and GSE143233. **i**–**k** The expression levels of PRIM1 in HCC cases or HCC cells with different sorafenib sensitivity in GSE63989, GSE 109211, and GSE62813. **l** The overall survival curves and recurrence free survival curves of HCC patients with high or low PRIM1 expression in entire cohort or stratified sub-groups of TCGA LIHC dataset. **m** The overall survival curves of HCC patients with high or low PRIM1 expression in entire cohort or stratified sub-groups of ICGC dataset. **P < 0.01; *P < 0.05

Firstly, we explored the expression features of PRIM1 in HCC by comprehensively analyzing multiple bioinformatic datasets. As shown in Fig. 2a–d, PRIM1 was overexpressed in HCC samples with advanced stages or grades, P53 mutation, and metastasis. Similarly, PRIM1 was significantly overexpressed in HCC samples of ICGC cohort (Fig. 2e). In addition, PRIM1 presented higher expression in advanced HCC cases than that of early HCC cases or pre-HCC cases (Fig. 2f–h). Moreover, overexpression of PRIM1 was also observed in HCC cases and HCC cells with sorafenib resistance in GSE63989, GSE109211, and GSE62813, respectively (Fig. 2i–k). Furthermore, we analyzed the prognostic value of PRIM1 in TCGA and ICGC datasets (Fig. 2l, m). High expression of PRIM1 indicated poor overall survival (OS) and recurrence free survival (RFS) of HCC patients in entire cohort or stratified sub-groups. Consistently, the univariate and multivariate analyses indicated that PRIM1 was an independent prognostic marker for OS and RFS of HCC patients (Additional file 1: Figure S1A–D).

### Expression features and prognostic value of PRIM1 in Nantong HCC cohort

**Fig. 3:**
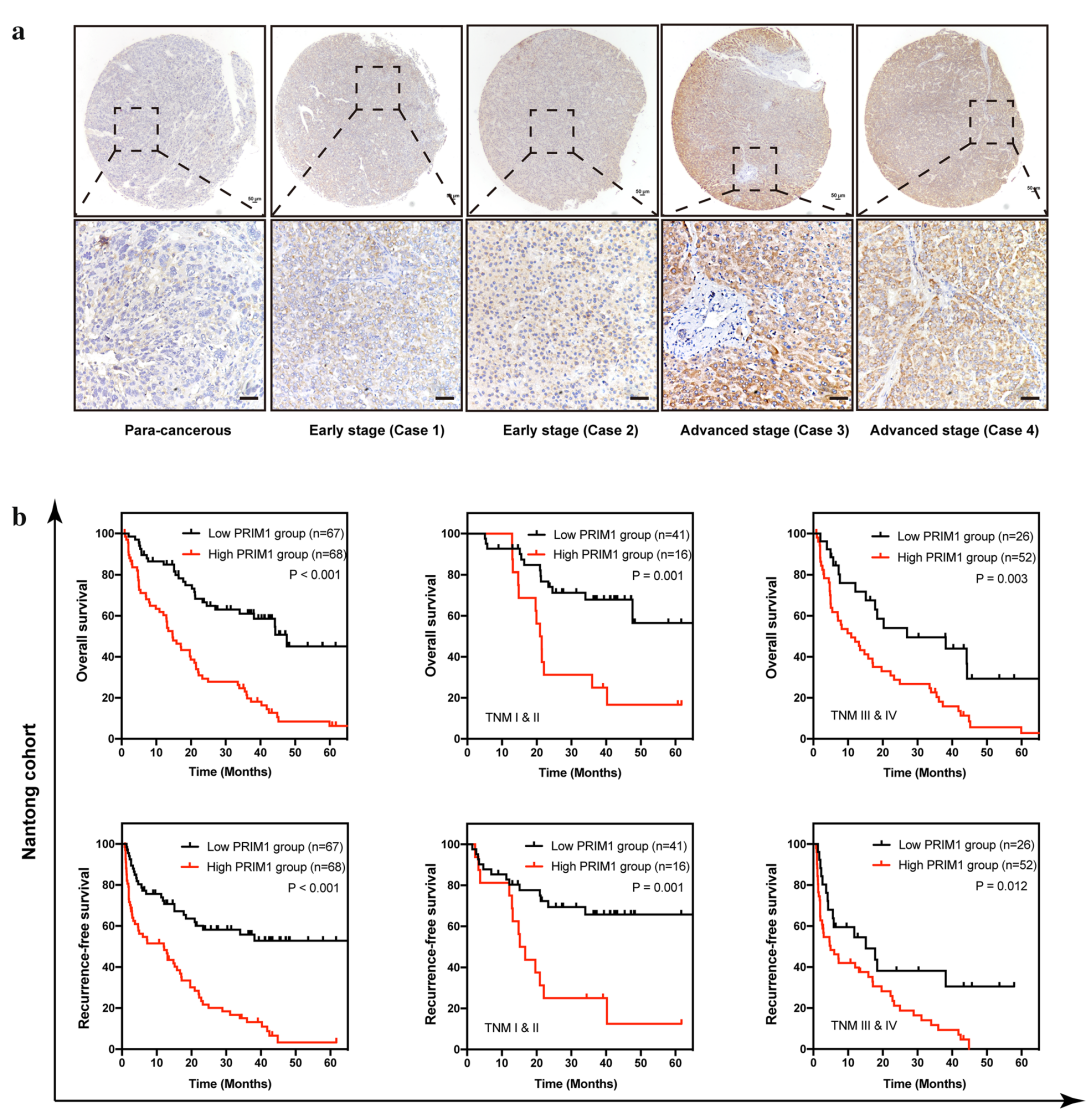
Investigating the expression features and prognostic values of PRIM1 in HCC by immunohistochemical analysis. **a** The representative immunohistochemical staining of PRIM1 in adjacent tissues and HCC tissues at different stages. **b** The overall survival curves and recurrence free curves of HCC patients with high or low PRIM1 expression in entire cohort or stratified sub-groups. Left panel, entire HCC cohort; middle panel, HCC patients at TNM stage I and II; right panel, HCC patients at TNM stage III and IV

Next, we conducted immunohistochemistry to detect the protein expression of PRIM1 in 135 self-paired tissues in Nantong HCC cohort. As shown in Fig. 3a, higher staining intensity of PRIM1 was observed in HCC cases compared with para-cancerous tissues. In addition, the expression of PRIM1 was dynamically elevated with HCC staging. Then we analyzed the correlation of PRIM1 with the clinical characteristics of the 135 HCC patients. High expression of PRIM1 was correlated with tumor size, Edmondson-Steiner grade, ascites, and advanced TNM stage (Table 2). Then, we performed the Kaplan–Meier analyses to discover the prognostic value of PRIM1 (Fig. 3b). The result demonstrated that patients with high expression of PRIM1 had shorter OS and RFS compared with cases with low PRIM1 expression. Interestingly, the stratification analysis suggested that overexpression of PRIM1 also indicated shorter survival in patients with early stages and advanced stages. In accordance, the univariate and multivariate analyses showed that PRIM1 was an independent predictor for the OS and RFS of HCC patents in Nantong cohort (Tables 3, 4).

**Table 2:** Correlation of PRIM1 expression with clinical features of 135 HCC patients

Characteristics		PRIM1 expression		*P* value
	Patients	Low	High	
Age (years)				
< 60	104	51	53	0.801
≥ 60	31	16	15	
Sex				
Male	113	55	58	0.614
Female	22	12	10	
AFP level				
< 20	30	19	11	0.089
≥ 20	105	48	57	
Tumor size (cm)				
< 3	26	21	5	*< 0.001*
≥ 3	109	46	63	
Edmondson-Steiner grade				
I + II	17	13	4	*0.018*
III + IV	118	54	64	
Ascites				
Absent	102	58	44	*0.003*
Present	33	9	24	
TNM stage				
I + II	57	41	16	*< 0.001*
III + IV	78	26	52	

*PRIM1* DNA primase subunit 1, *TNM* tumor-node-metastasis

**Table 3:** Univariate and multivariate analyses of the overall survival in 135 HCC cases

Parameters	Univariate analysis			Multivariate analysis		
	HR	95% CI	*P*	HR	95% CI	*P*
PRIM1 expression						
High vs. low	3.271	2.080–5.146	*< 0.001*	2.042	1.248–3.343	*0.005*
Age (years)						
< 60 vs. ≥ 60	0.905	0.548–1.493	0.695			
Sex						
Male vs. female	0.920	0.517–1.636	0.777			
AFP level						
Positive vs. negative	1.722	1.012–2.932	*0.043*	1.564	0.912–2.648	0.104
Tumor size (cm)						
< 3 vs. ≥ 3	3.707	1.852–7.419	< *0.001*	2.075	0.968–4.449	0.061
Edmondson-Steiner grade						
I + II vs. III + IV	2.404	1.158–4.990	*0.015*	1.643	0.766–3.522	0.202
Ascites						
Absent vs. present	2.530	1.620–3.951	< *0.001*	2.377	1.497–3.773	*< 0.001*
TNM stage						
I + II vs. III + IV	2.697	1.701–4.275	< *0.001*	1.712	1.023–2.862	*0.041*

*PRIM1* DNA primase subunit 1, *TNM* tumor-node-metastasis

**Table 4:** Univariate and multivariate analyses of the recurrence-free survival in 135 HCC cases

Parameters	Univariate analysis			Multivariate analysis		
	HR	95% CI	*P*	HR	95% CI	*P*
PRIM1 expression						
High vs. low	3.283	2.083–5.173	*< 0.001*	1.733	1.038–2.893	*0.036*
Age (years)						
< 60 vs. ≥ 60	0.857	0.520–1.414	0.545			
Sex						
Male vs. female	1.026	0.579–1.819	0.930			
AFP level						
Positive vs. negative	1.520	0.894–2.586	0.119			
Tumor size (cm)						
< 3 vs. ≥ 3	3.311	1.654–6.627	*< 0.001*	1.849	0.873–3.915	0.108
Edmondson-Steiner grade						
I + II vs. III + IV	2.172	1.048–4.503	*0.032*	1.878	0.882–3.997	0.102
Ascites						
Absent vs. present	2.405	1.541–3.754	*< 0.001*	2.431	1.516–3.898	*< 0.001*
TNM stage						
I + II vs. III + IV	3.024	1.903–4.805	*< 0.001*	2.201	1.306–3.707	*0.003*

*PRIM1* DNA primase subunit 1, *TNM* tumor-node-metastasis

### PRIM1 enhanced the aggressive behaviors and facilitated EMT phenotypes

**Fig. 4:**
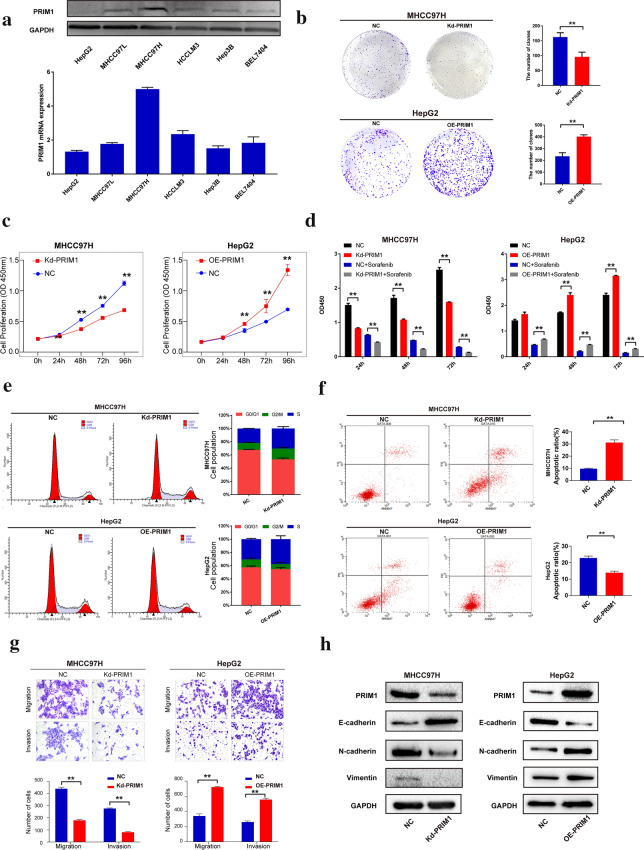
PRIM1 facilitated aggressive phenotypes and EMT of HCC cells. **a** The protein and mRNA expression of PRIM1 in HCC cell lines were detected by western blotting and RT-qPCR, respectively. **b** The colony formation of PRIM1-silenced MHCC97H and PRIM1-overexpressed HepG2. **c** Following transfection of Kd-PRIM1 or OE-PRIM1 plasmid, the proliferation of HCC cells was detected by CCK-8 assay. **d** The sorafenib sensitivity was detected in PRIM1-silenced MHCC97H or PRIM1-overexpressed HepG2 cells compared with NC group. **e**, **f** Following transfection of Kd-PRIM1 or OE-PRIM1 plasmid, the cell cycle and apoptosis were detected by flowcytometry. **g** The migration and invasion of MHCC97H or HepG2 cells in each group were detected by transwell assay. **h** The expression of EMT markers were examined by western blotting with PRIM1 overexpression or downregulation. **P < 0.01; *P < 0.05

Given the expression features and clinical significance of PRIM1, we further investigated the effects of PRIM1 on the biological behaviors of the HCC cells. As shown in Fig. 4a, differential expression of PRIM1 was observed in HCC cell lines at protein and mRNA levels. Then we chose MHCC97H and HepG2 to conduct Loss-of-function and gain-of-function studies, respectively. As elucidated in Fig. 4b, c, knockdown of PRIM1 significantly inhibited the colony formation, proliferation of MHCC97H cells, while ectopic expression of PRIM1 promoted colony formation and cell proliferation of HepG2 cells. Furthermore, PRIM1 could enhance the resistance of HCC cells against sorafenib (Fig. 4d). In addition, silencing or overexpressing PRIM1 increased or decreased the G2/M arrest and apoptosis of MHCC97H or HepG2 cells, respectively (Fig. 4e, f). Remarkably, knockdown or overexpression of PRIM1 significantly enhanced or reduced the migration and invasion capacity of HCC cells (Fig. 4g). Consistently, PRIM1 could enhance the expression of Vimentin and *N*-cadherin and downregulate the expression of E-cadherin, suggesting its roles in promoting epithelial-mesenchymal transition (EMT) of HCC cells (Fig. 4h). Thus, PRIM1 might facilitate the aggressive behaviors of HCC cells.

### PRIM1 activated the AKT/MTOR signaling of HCC cells

**Fig. 5:**
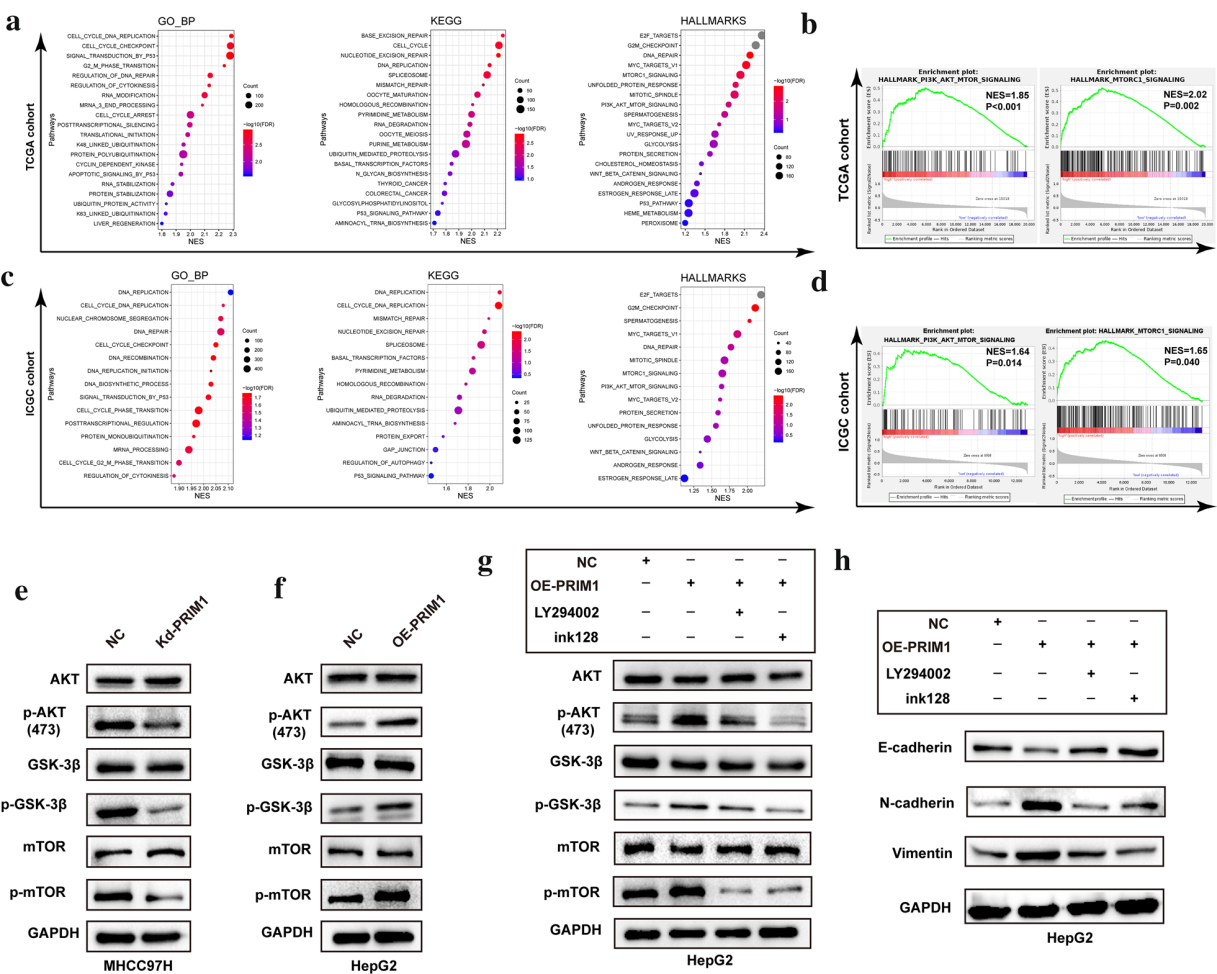
PRIM1 activated AKT/mTOR signaling of HCC cells. **a** Gene sets enrichment analysis (GSEA) of PRIM1 in TCGA LIHC dataset. **b** GSEA of PRIM1 in ICGC dataset. **c** The enrichment plot of PI3K/AKT/mTOR signaling and mTORC1 in TCGA LIHC dataset. **d** The enrichment plot of PI3K/AKT/mTOR signaling and mTORC1 in ICGC dataset. **e**, **f** The expression of PI3K/AKT/mTOR signaling markers was detected by western blotting, following PRIM1 knockdown or overexpression in HCC cells. **g** The expression of PI3K/AKT/mTOR signaling markers in PRIM1-overexpressed HepG2 cells treated with LY294002 or ink128. **h** The expression of EMT markers in PRIM1-overexpressed HepG2 cells treated with LY294002 or ink128. NES, normalized enrichment score

To further discover the underlying mechanisms, we conducted GSEA and simultaneously in the TCGA cohort and ICGC cohort (Fig. 5a, b). Similar with ORA (Additional file 2: Figure S2), PRIM1 was implicated in various tumor-related biological processes, including cell cycle, DNA replication, DNA repair, and autophagy. In addition, PRIM1 might regulate the activity of mTORC1 signaling and PI3K/AKT/mTOR signaling, which were previously defined as canonical metastasis and proliferation related pathways (Fig. 5c, d). Consistently, depletion of PRIM1 inhibited the expression of p-AKT, p-GSK-3β, and p-mTOR of MHCC97H cells (Fig. 5e). Ectopic expression of PRIM1 enhanced the expression of these markers, while AKT inhibitor LY294002 or mTOR inhibitor ink128 significantly abrogated the activation of the PI3K/AKT/mTOR signaling (Fig. 5f, g). Moreover, LY294002 or ink128 reversed the enhancement of EMT markers induced by exogenous PRIM1 (Fig. 5h). Thus, PRIM1 might promote the aggressive behaviors of HCC cells by activating PI3K/AKT/mTOR signaling.

### PRIM1 promoted P53 ubiquitination and degradation

**Fig. 6:**
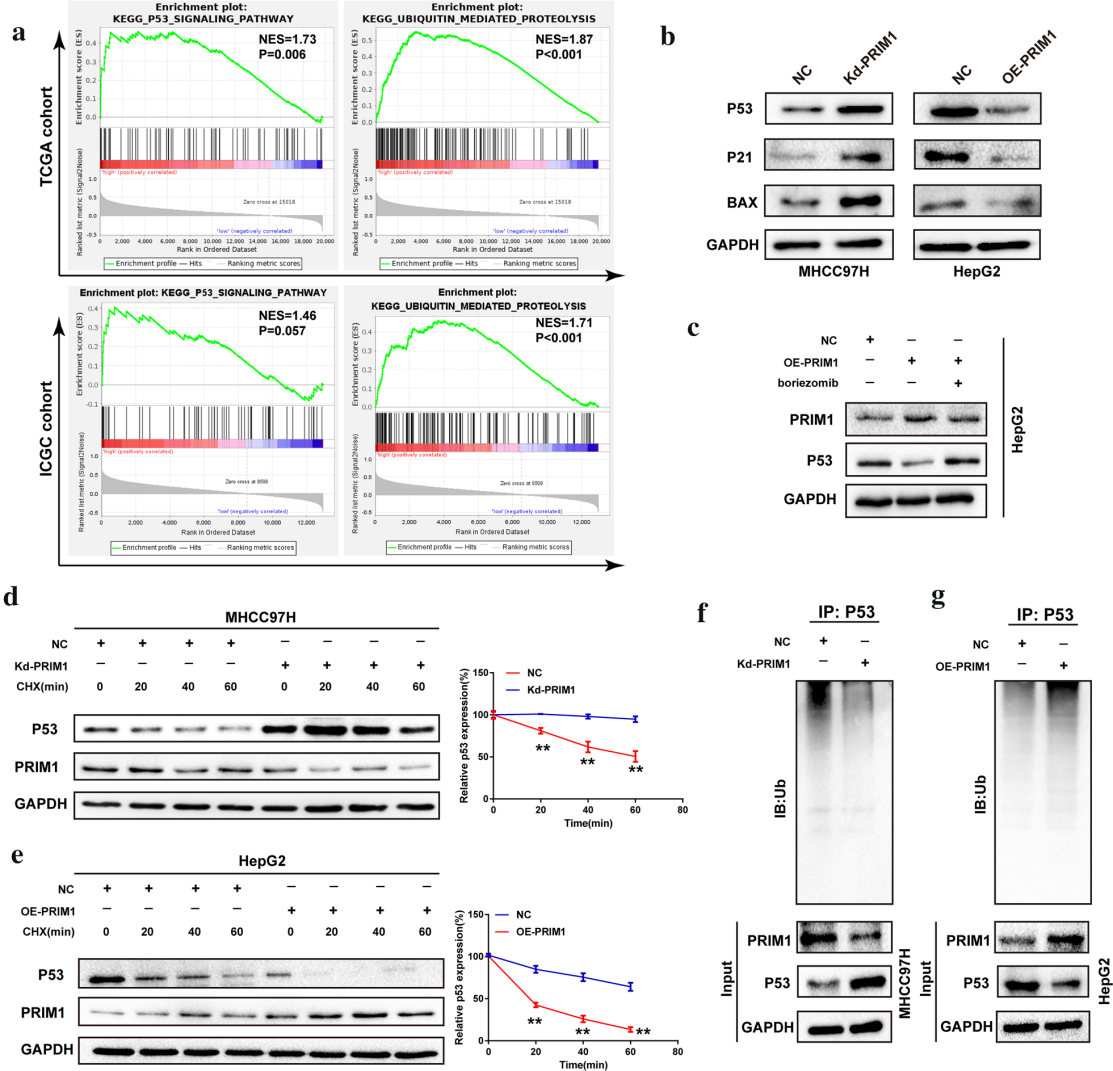
PRIM1 downregulated P53 in a ubiquitination-dependent manner. **a** The enrichment plot of P53 signaling and ubiquitin mediated proteolysis in TCGA and ICGC datasets. **b** The expression of P53 signaling of HCC cells with PRIM1 knockdown or overexpression. **c** The expression of P53 expression in PRIM1-overexpressed HepG2 cells treated with boriezomib. **d** Expression of P53 in MHCC97H cells transfected with or without Kd-PRIM1 and treated with CHX (100 μM) for the indicated times. **e** Expression of P53 in HepG2 cells transfected with or without OE-PRIM1 and treated with CHX for the indicated times. **f** Ubiquitination of P53 in PRIM1-silenced MHCC97H cells. **g** Ubiquitination of P53 in PRIM1-overexpressed HepG2 cells

P53 is a vital tumor-suppressive signaling, for which ubiquitination is a common post-transcriptional modification. GSEA indicated that PRIM1 might be involved in regulating P53 signaling and ubiquitin mediated proteolysis (Fig. 6a). Thus, we further explored the regulatory effects of PRIM1 on P53 signaling in HCC cells. Knockdown of PRIM1 upregulated the expression of P53, P21 and BAX (Fig. 6b). In contrast, PRIM1 overexpression significantly inactivated P53 signaling of HepG2 cells, which was restored by protease inhibitor boriezomib (Fig. 6c). Moreover, silencing PRIM1 alleviated the degradation of P53 induced by CHX. Conversely, exogenous PRIM1 accelerated the degradation of P53 (Fig. 6d, e). Furthermore, we discovered the correlation of PRIM1 mediated P53 degradation with ubiquitination. As proposed, the weakened ubiquitination of P53 from the P53-lysates immunocomplex was detected in Kd-PRIM1 transfected MHCC97H cells compared with NC group following elevated P53 protein expression (Fig. 6f). Conversely, the enhanced ubiquitination of p53 was observed in the p53-lysates immunocomplex in OE-PRIM1 transfected HepG2 cells compared with NC groups following decreased P53 expression (Fig. 6g). Taken together, PRIM1 promoted P53 ubiquitination and degradation of HCC cells.

### PRIM1 facilitated P53 ubiquitination by upregulating UBE2C

**Fig. 7:**
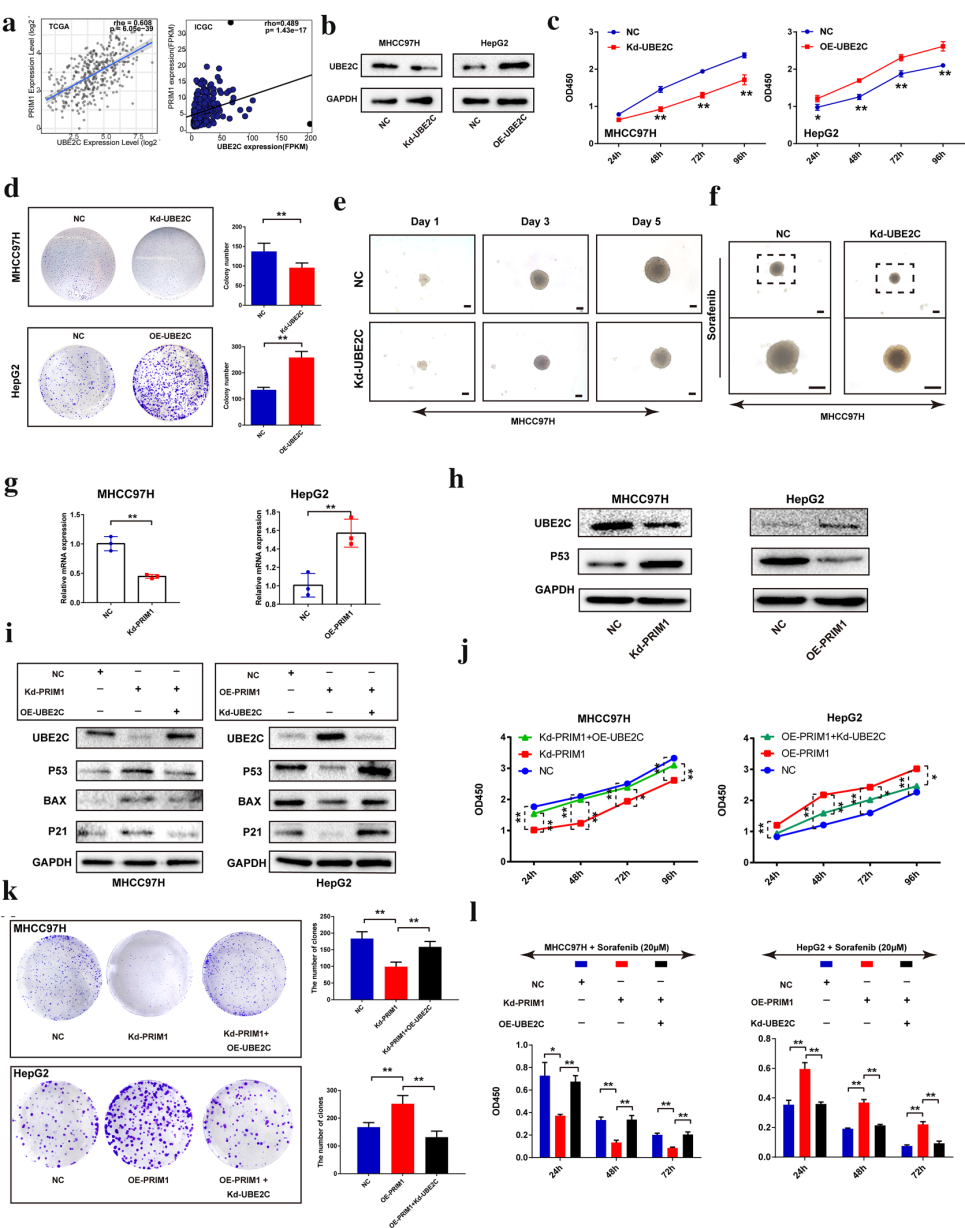
PRIM1 promoted P53 degradation via up-regulating UBE2C in HCC cells. **a** the Spearman correlation of PRIM1 with UBE2C in TCGA and ICGC datasets. **b** MHCC97H and HepG2 were transfected with Kd-UBE2C or OE-UBE2C plasmids, respectively. **c** The proliferation of HCC cells was detected by CCK-8 assay. **d** The colony formation of HCC cells in each group. **e** The growth of the 3D spheres derived from MHCC97H in each group during day 1–5. **f** The MHCC97H-derived spheroids in different group treated with sorafenib. **g** The mRNA levels of UBE2C with PRIM1 knockdown or overexpression in MHCC97H or HepG2 cells. **h** The protein levels of UBE2C and P53 with PRIM1 depletion or overexpression in MHCC97H or HepG2 cells. **i** The protein levels of P53 signaling markers in MHCC97H or HepG2 cells transfected with different plasmids. **j** The proliferation of MHCC97H or HepG2 cells transfected with different plasmids was detected by CCK-8. **k** The colony formation of MHCC97H or HepG2 cells in each group. **l** The sensitivity of MHCC97H or HepG2 cells transfected with different plasmids to sorafenib treatment. **P < 0.01; *P < 0.05

Next, we further investigated the potential mechanisms of PRIM1-mediated P53 ubiquitination by screening the genes enriched in the item “ubiquitin mediated proteolysis”. P53 was previously defined as a substrate of one of the top genes Ubiquitin Conjugating Enzyme E2 C (UBE2C) [17]. Firstly, we investigated the correlation of UBE2C with PRIM1 and expression features of UBE2C in bioinformatic datasets. As shown in Fig. 7a, PRIM1 mRNA level was positively correlated with UBE2C in both of TCGA and ICGC datasets. Similar with PRIM1, overexpression of UBE2C was observed in HCC tissues with P53 mutation or at advanced grades (Additional file 3: Figure S3A–D). Moreover, the expression level of UBE2C was higher in high dysplastic liver nodules and sorafenib resistant HepG2 cells (Additional file 3: Figure S3E and F**)**. Additionally, high expression of UBE2C indicated poor survival of HCC patients in TCGA (Additional file 4: Figure S4A) and ICGC datasets (Additional file 4: Figure S4B). Then we conducted functional validations regarding the effects of UBE2C. Silencing UBE2C significantly repressed the proliferation and colony formation of MHCC97H cells; In contrast, overexpression of UBE2C enhanced the aggressive behaviors of HepG2 cells (Fig. 7c, d). Moreover, 3D spheroid model indicated that PRIM1 facilitated tumor growth and chemoresistance of sorafenib (Fig. 7e, f; Additional file 5: Figure S5). We hypothesized that UBE2C might serve as an important oncogene that participated in regulating PRIM1-mediated P53 ubiquitination and related phenotypes of HCC. As shown in Fig. 7g, h, knockdown of PRIM1 inhibited UBE2C expression of MHCC97H cells in both of the mRNA and protein levels, while overexpressing PRIM1 enhanced the expression of UBE2C of HepG2 cells. Furthermore, overexpression or depletion of UBE2C abolished the alterations of P53 signaling induced by PRIM1 in HCC cells (Fig. 7i). Consistently, exogenous UBE2C restored proliferation, colony formation, and sorafenib resistance of MHCC97H cells inhibited by PRIM1 repression. In addition, knockdown of UBE2C abrogated the enhancement of these aggressive phenotypes following PRIM1 overexpression (Fig. 7j–l). Thus, PRIM1 might promote the aggressive behaviors via UBE2C-mediated P53 ubiquitination.

### PRIM1 enhanced tumor growth in the xenograft model and 3D spheroid model

**Fig. 8:**
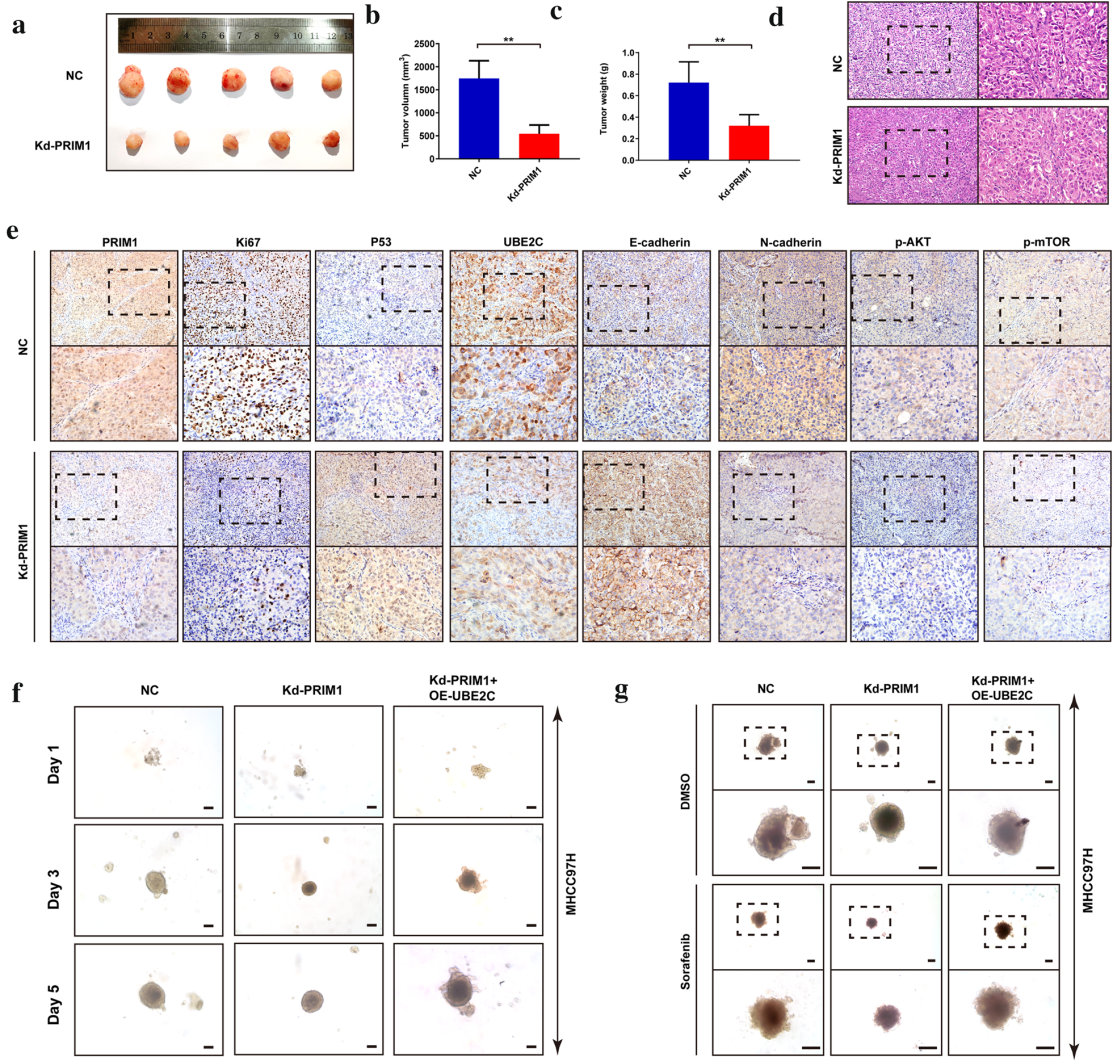
PRIM1 enhanced tumor growth in xenograft model and 3D spheroid model. **a** The MHCC97H-derived xenograft tumors of NC group and Kd-PRIM1 group. **b**, **c** The volume and weight of MHCC97H-derived xenograft tumors in NC group and Kd-PRIM1 group. **d** The representative H&E staining of the xenograft tumors in each group. **e** The representative immunohistochemical staining (PRIM1, P53, UBE2C, Ki67, E-cadherin, N-cadherin, P-AKT, and P-mTOR) of the xenograft tumors. **f** The growth of the 3D spheroids derived from MHCC97H in each group in day 1–5. **g** The MHCC97H-derived spheroids in different group treated with sorafenib. **P < 0.01

Ultimately, we validated the roles of PRIM1 in vivo and 3D models. As shown in Fig. 8a–c, the mean volume and weight of PRIM1-silenced MHCC97H-derived xenograft tumors were significantly less than these of NC group. In addition, the H&E staining showed that knockdown of PRIM1 alleviated the typically histological features of xenografts (Fig. 8d). Further immunohistochemical staining demonstrated that silencing PRIM1 significantly decreased the expression of UBE2C, Ki67, N-cadherin, P-AKT, and P-mTOR of the xenograft tumor tissues, with the increase of E-cadherin and P53 expression (Fig. 8e). Next, we constructed 3D spheroid model to further investigate the role of PRIM1. As elucidated in Fig. 8f, depletion of PRIM1 impeded the growth of the 3D spheroids derived from MHCC97H cells in day 1–5, while ectopic expression of UBE2C restored the growth of the spheroids. Besides, PRIM1 silenced MHCC97H-derived spheroids presented higher sorafenib sensitivity than the NC group. Further exogenous UBE2C enhanced the resistance of the spheroids against sorafenib (Fig. 8g). In consistent, silencing UBE2C abrogated the enhancement of growth and sorafenib resistance induced by PRIM1 overexpression in HepG2-derived spheroids (Additional file 6: Figure S6A and B). These evidences suggested that PRIM1 might contributed to tumor growth and sorafenib resistance.

## Discussion

Various hub genes and signaling pathways maintain the intracellular homeostatic status. The abnormal expression of oncogenes is critical to hepatocarcinogenesis and HCC progression, which may be key targets with the potential to improve HCC treatment [18]. A better understanding of hub genes that involve in HCC carcinogenesis will contribute to identifying potentially therapeutic targets [19]. Firstly, we identified DEGs between HCC and normal liver tissues in GEO and TCGA datasets, which were implicated in multiple cell cycle and DNA replication related terms. Of them, PRIM1 was one of the top genes associated with most of the enriched pathways and predicted poor prognostic of HCC patients. In addition, the roles of PRIM1 in phenotypic manifestations of HCC and the underlying molecular mechanisms have not been well elucidated. Therefore, we focused on the investigation of PRIM1 from the aspects of expression profiling, clinical significance, effects on aggressive behaviors, and molecular mechanisms in HCC.

Although emerging studies have indicated the potential roles of PRIM1 in the malignant behaviors of breast cancer [13], the expression features and functions of PRIM1 in HCC remain unclear. In this study, elevated mRNA and protein expression of PRIM1 were determined in HCC tissues from various bioinformatic datasets and tissue microarray. Overexpression of PRIM1 was correlated with aggressive clinical characteristics and poor survival of HCC patients. In the current study, the prognostic value of PRIM1 was validated in TCGA, ICGC and local Nantong cohorts, suggesting that it might be an attractive marker for HCC. Apart from its clinical implications, loss-of-function and gain-of-function studies suggested the contributing roles of PRIM1 in the malignant phenotypes including proliferation, colony formation, invasion and sorafenib resistance, and EMT processes. To further explore the mechanisms, we conducted GSEA based on the TCGA and ICGC datasets. Apart from the enrichment in the biological processes such as cell cycle and DNA proliferation, the GSEA suggested that PRIM1 might be implicated in AKT/GSK-3β and mTOR signaling. Both in vitro and in vivo studies have demonstrated that PI3K/AKT/mTOR signaling plays key roles in regulating HCC initiation and progression [20]. It regulates proliferation, cancer stem cell properties, and invasion and migration of HCC via phosphorylating its downstream effectors [21]. As expected, PRIM1 could regulated the activity of PI3K/AKT/mTOR signaling. The inhibitors of AKT or mTOR reversed the modulation induced by PRIM1 overexpression. Moreover, the inhibitors also restored the activation of EMT markers induced by exogenous PRIM1. Therefore, it was supposed that PRIM1 positively regulated the activity of PI3K/AKT/mTOR signaling and its mediated malignant behaviors. A recent work using CRISPR-based-genome-wide screening determined PRIM1 as a robust regulator of cAMP-PKA signaling. Further validation also confirmed that deletion of PRIM1 significantly blunted the activation of cAMP signaling via multi-stimuli [22]. As an important second messenger, cAMP is competent in multiple signaling transduction and pathway linking. For example, cAMP-PKA axis has been reported to facilitate PI3K-AKT and subsequent mTORC activation [23]. Hence, PRIM1 may regulate PI3K-AKT-mTOR partially through ensuring the activity of cAMP-PKA cascade. However, it is the speculation of the PRIM1-mediated mechanism, and the explicit regulatory network should be elucidated by further investigations.

P53 signaling is the most intensively studied tumor-suppressive hallmark that modulates multiple biological processes, such as proliferation, senescence, and apoptosis [24]. Gao et al. revealed that P53 mutation occurs in almost 60% HBV-related HCC patients, suggesting that mutation of the P53 gene abrogates the P53 tumor-suppressor function to deteriorate hepatocarcinogenesis [25]. Thus, it is an attractive strategy to reactivate P53 signaling in HCC patients with P53 mutation. According to the bioinformatic analyses in TCGA and ICGC datasets, PRIM1 might be implicated in the regulation of P53 signaling. Further molecular validation found that PRIM1 downregulated the expression of P53 and its downstream genes. Then we further discovered the molecular mechanisms of PRIM1 mediated P53 repression. Based on the GSEA, PRIM1 might participate in the ubiquitin mediated proteolysis. The protease inhibitor boriezomib reversed the inhibition of P53 induced by PRIM1 overexpression. Thus, we hypothesized that PRIM1 inactivated P53 via ubiquitination-mediated degradation. With the treatment of CHX, PRIM1 overexpression enhanced the attenuation of P53 protein expression of MHCC97H cells, while silencing PRIM1 alleviated the P53 degradation induced by CHX. PRIM1 silencing or overexpression decreased or enhanced p53 ubiquitination with the increase or decrease of p53 protein expression, respectively. It was consistent with our hypothesis that PRIM1 de-stabilized P53 in a ubiquitination-dependent manner.

Ubiquitination is a crucial post-translational modification for protein stability. By searching the enriched genes in GSEA analysis, UBE2C was selected for further validation due to a previous study that suggested P53 protein as its ubiquitination substrate [17]. Consistently, UBE2C, as a member of ubiquitin-conjugating enzyme (E2) family, has been reported to be abnormally expressed in HCC and involved in its initiation and progression [26, 27]. Similar with the expression features of PRIM1, UBE2C was overexpressed in HCC tissues at advanced grades or sorafenib-resistant HCC cells. In both of the TCGA datasets and ICGC datasets, PRIM1 presented positive correlation with UBE2C in HCC samples. Overexpression or knockdown of UBE2C abrogated the alteration of P53 expression induced by PRIM1. In addition, silencing UBE2C inhibited the proliferation, colony formation, tumor spheroid growth, and sorafenib resistance induced by exogenous PRIM1. Therefore, we drew the hypothesis that PRIM1 promoted these aggressive phenotypes partially through UBE2C-mediated P53 degradation. Interestingly, Luo YD et al. recently noted that P53 haploinsufficiency and mTOR signaling activation identified a subset of aggressive HCC patients [28]. In the current study, we found that depletion of PRIM1 reactivated P53 signaling and decreased the activity of AKT/mTOR signaling. Based on such observation, targeting PRIM1 may provide a new strategy for treating aggressive subtype of HCC.

## Conclusion

In conclusion, this study identified PRIM1 as a pivotal regulator that deteriorated the aggressive phenotypes of HCC cells by modulating AKT/mTOR signaling and UBE2C-mediated P53 inactivation, suggesting that PRIM1 might serve as a therapeutic target in the prevention and treatment of HCC.

## Supplementary Information

**Additional file 1: Figure S1. **The forest plots of the PRIM1 in TCGA LIHC dataset. (A and B) The forest plots of the univariate and multivariate analyses for the OS of HCC cases in TCGA datasets. (C and D) The forest plots of the univariate and multivariate analyses for the RFS of HCC cases in TCGA datasets.

**Additional file 2: Figure S2.** The ORA analyses of the PRIM1 in TCGA LIHC dataset. (A) The significantly positively- or negatively- correlated genes with PRIM1 in the TCGA LIHC dataset. (B) The potential PRIM1-mediated functions and pathways predicted by ORA in the TCGA LIHC dataset.

**Additional file 3: Figure S3.** The expression features of UBE2C in bioinformatic databases. (A) The expression levels of UBE2C in HCC tissues and normal liver tissues in ICGC dataset. (B and C) The expression levels of UBE2C in HCC patients with TP53 mutation and different grades in TCGA LIHC dataset. (D and E) The expression levels of UBE2C in HCC cases and pre-HCC cases in GSE89377. (F) The expression levels of UBE2C in HCC cases or sorafenib sensitive or resistant HepG2 cells in GSE62813. **, P<0.01; *, P<0.05.

**Additional file 4: Figure S4.** UBE2C indicated poor survival of HCC patients. (A) The overall survival curves and recurrence free curves of HCC patients with high or low PRIM1 expression in entire cohort or sorafenib-treated sub-groups in TCGA LIHC dataset. (B) The overall survival curves of HCC patients with high or low PRIM1 expression in entire cohort or sub-groups in ICGC dataset.

**Additional file 5: Figure S5.** UBE2C enhanced tumor growth and sorafenib resistance in HepG2-derived spheroids model. (A) The growth of the 3D spheroids derived from HepG2 in each group during Day1-5. (B) The HepG2-derived spheroids in different group treated with sorafenib.

**Additional file 6: Figure S6.** PRIM1 enhanced tumor growth and sorafenib resistance in HepG2-derived spheroids model. (A) The growth of the 3D spheroids derived from HepG2 in each group from Day1-5. (B) The HepG2-derived spheroids in different group treated with sorafenib.

## Data Availability

All data generated or analyzed during this study are included in this published article. All of the data and material in this study are available when requested.

## Abbreviations

HCC: Hepatocellular carcinoma
HBV: Hepatitis B virus
HCV: Hepatitis C virus
PRIM1: DNA primase subunit 1
NCBI: National Center for Biotechnology Information
GEO: Gene expression omnibus
TCGA-LIHC: The cancer genome atlas liver hepatocellular carcinoma
ICGC: International Cancer Genome Consortium
GSEA: Gene set enrichment analysis
ORA: Over-representation enrichment analysis
CCK-8: Cell counting kit-8 assay
RIPA: Radioimmunoprecipitation assay buffer
PI: Propidium iodide
SDS: Sodium dodecyl sulfate
PVDF: Polyvinylidene difluoride
HPR: Horseradish peroxidase
ECL: Enhanced chemiluminescence
GAPDH: Glyceraldehyde phosphate dehydrogenase
GO: Gene ontology
CCs: Cellular components
MFs: Molecular functions
OS: Overall survival
RFS: Recurrence free survival
UBE2C: Ubiquitin conjugating enzyme E2 C
