# Supplementary material for: DNA primase subunit 1 deteriorated progression of hepatocellular carcinoma by activating AKT/mTOR signaling and UBE2C-mediated P53 ubiquitination
Source: Cell Biosci. 2021 Feb 23;11:42. doi: 10.1186/s13578-021-00555-y (PMC7903777; doi:10.1186/s13578-021-00555-y)
Supplement: Supplementary file 1 — Additional file 1: Figure S1. The forest plots of the PRIM1 in TCGA LIHC dataset. (A and B) The forest plots of the univariate and multivariate analyses for the OS of HCC cases in TCGA datasets. (C and D) The forest plots of the univariate and multivariate analyses for the RFS of HCC cases in TCGA datasets. [file 13578_2021_555_MOESM1_ESM.pdf]

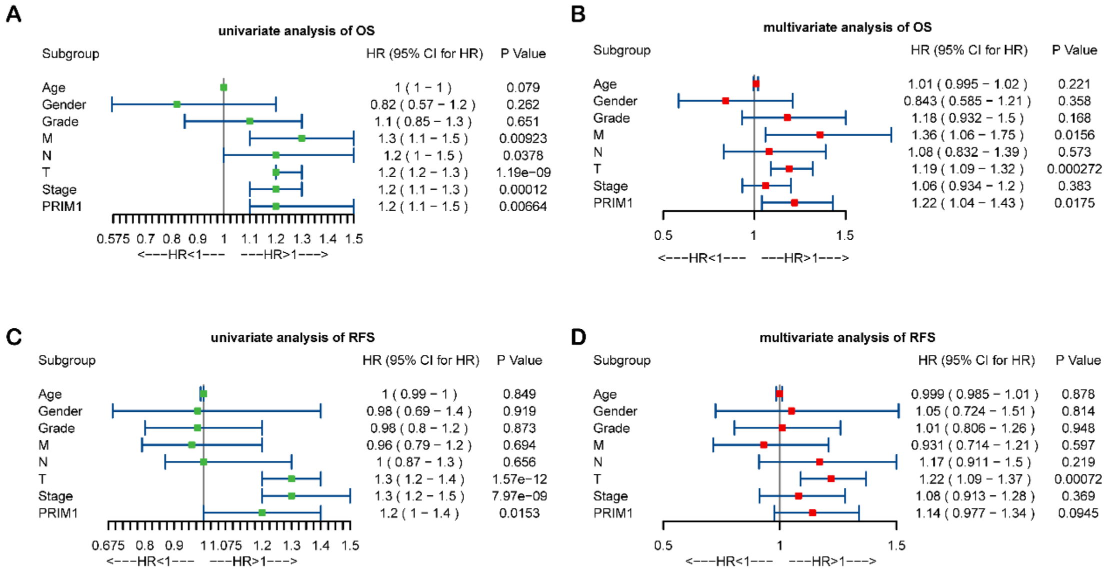

Figure S1. The forest plots of the PRIM1 in TCGA LIHC dataset.

(A and B) The forest plots of the univariate and multivariate analyses for the OS of HCC cases in TCGA datasets. **(C and D)** The forest plots of the univariate and multivariate analyses for the RFS of HCC cases in TCGA datasets.
