# Supplementary material for: DNA primase subunit 1 deteriorated progression of hepatocellular carcinoma by activating AKT/mTOR signaling and UBE2C-mediated P53 ubiquitination
Source: Cell Biosci. 2021 Feb 23;11:42. doi: 10.1186/s13578-021-00555-y (PMC7903777; doi:10.1186/s13578-021-00555-y)
Supplement: Supplementary file 2 — Additional file 2: Figure S2. The ORA analyses of the PRIM1 in TCGA LIHC dataset. (A) The significantly positively- or negatively- correlated genes with PRIM1 in the TCGA LIHC dataset. (B) The potential PRIM1-mediated functions and pathways predicted by ORA in the TCGA LIHC dataset. [file 13578_2021_555_MOESM2_ESM.pdf]

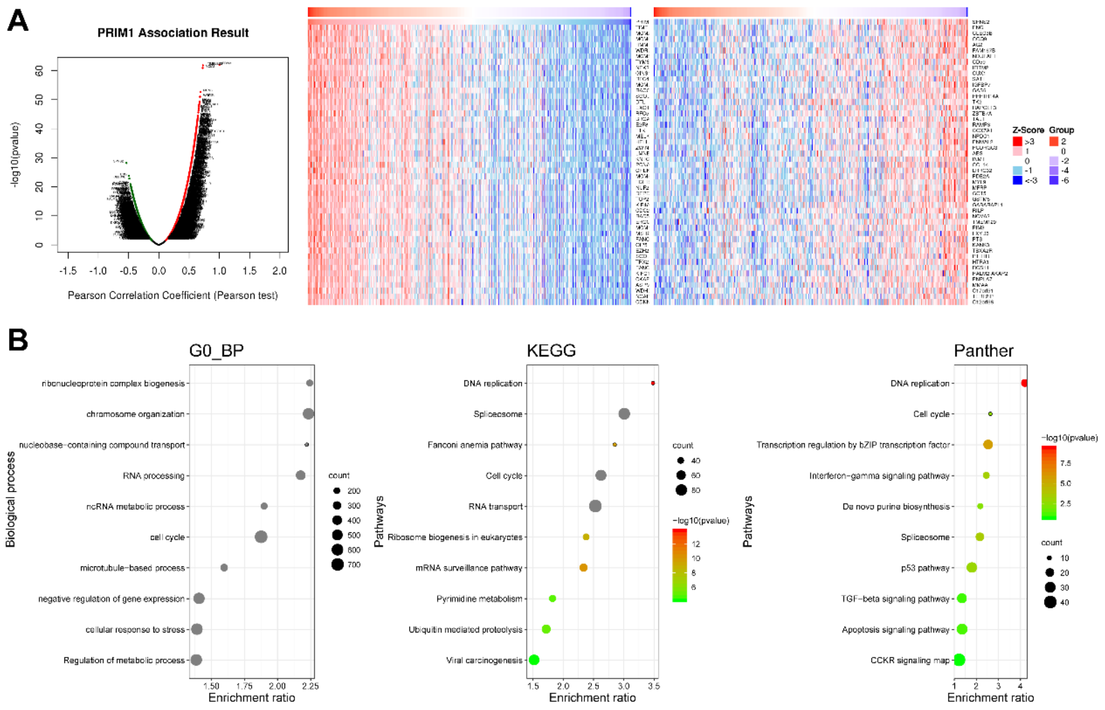

Figure S2. The ORA analyses of the PRIM1 in TCGA LIHC dataset.

(A) The significantly positively- or negatively- correlated genes with PRIM1 in the TCGA LIHC dataset. **(B-D)** The potential PRIM1-mediated functions and pathways predicted by ORA in the TCGA LIHC dataset.
