# Supplementary material for: DNA primase subunit 1 deteriorated progression of hepatocellular carcinoma by activating AKT/mTOR signaling and UBE2C-mediated P53 ubiquitination
Source: Cell Biosci. 2021 Feb 23;11:42. doi: 10.1186/s13578-021-00555-y (PMC7903777; doi:10.1186/s13578-021-00555-y)
Supplement: Supplementary file 3 — Additional file 3: Figure S3. The expression features of UBE2C in bioinformatic databases. (A) The expression levels of UBE2C in HCC tissues and normal liver tissues in ICGC dataset. (B and C) The expression levels of UBE2C in HCC patients with TP53 mutation and different grades in TCGA LIHC dataset. (D and E) The expression levels of UBE2C in HCC cases and pre-HCC cases in GSE89377. (F) The expression levels of UBE2C in HCC cases or sorafenib sensitive or resistant HepG2 cells in GSE62813. **, P<0.01; *, P<0.05. [file 13578_2021_555_MOESM3_ESM.pdf]

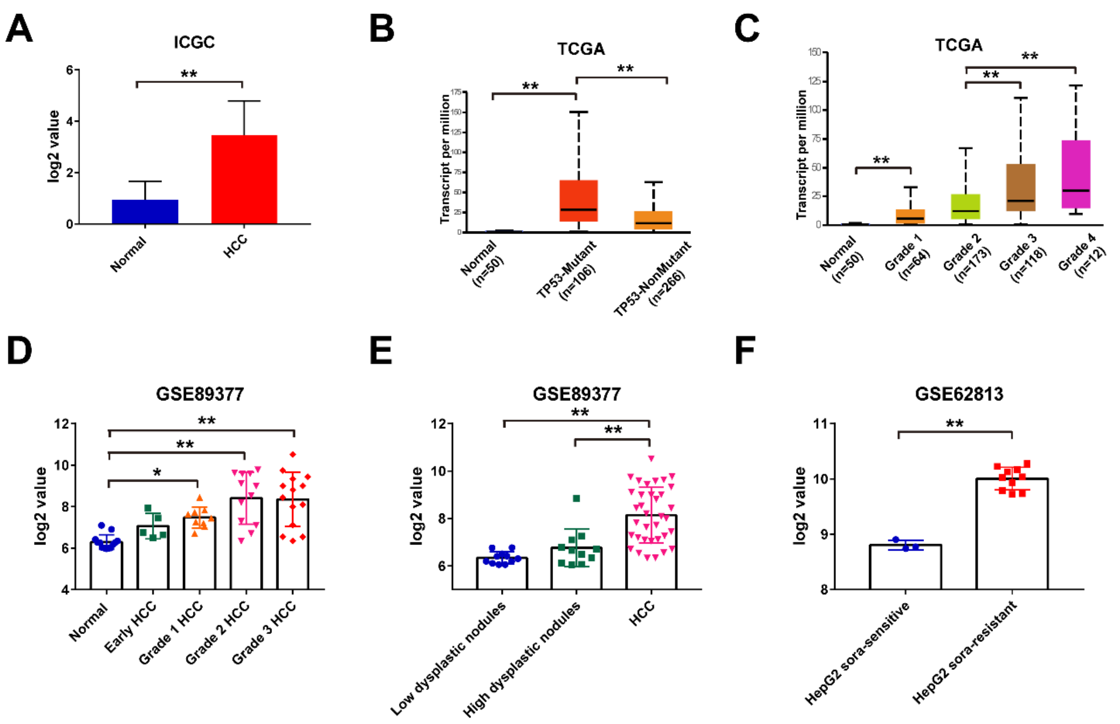

Figure S3. The expression features of UBE2C in bioinformatic databases. (A) The expression levels of UBE2C in HCC tissues and normal liver tissues in ICGC dataset. (B and C) The expression levels of UBE2C in HCC patients with TP53 mutation and different grades in TCGA LIHC dataset. **(D and E)** The expression levels of UBE2C in HCC cases and pre-HCC cases in GSE89377. **(F)** The expression levels of UBE2C in HCC cases or sorafenib sensitive or resistant HepG2 cells in GSE62813. **, P<0.01; *, P<0.05.
