# Supplementary material for: DNA primase subunit 1 deteriorated progression of hepatocellular carcinoma by activating AKT/mTOR signaling and UBE2C-mediated P53 ubiquitination
Source: Cell Biosci. 2021 Feb 23;11:42. doi: 10.1186/s13578-021-00555-y (PMC7903777; doi:10.1186/s13578-021-00555-y)
Supplement: Supplementary file 4 — Additional file 4: Figure S4. UBE2C indicated poor survival of HCC patients. (A) The overall survival curves and recurrence free curves of HCC patients with high or low PRIM1 expression in entire cohort or sorafenib-treated sub-groups in TCGA LIHC dataset. (B) The overall survival curves of HCC patients with high or low PRIM1 expression in entire cohort or sub-groups in ICGC dataset. [file 13578_2021_555_MOESM4_ESM.pdf]

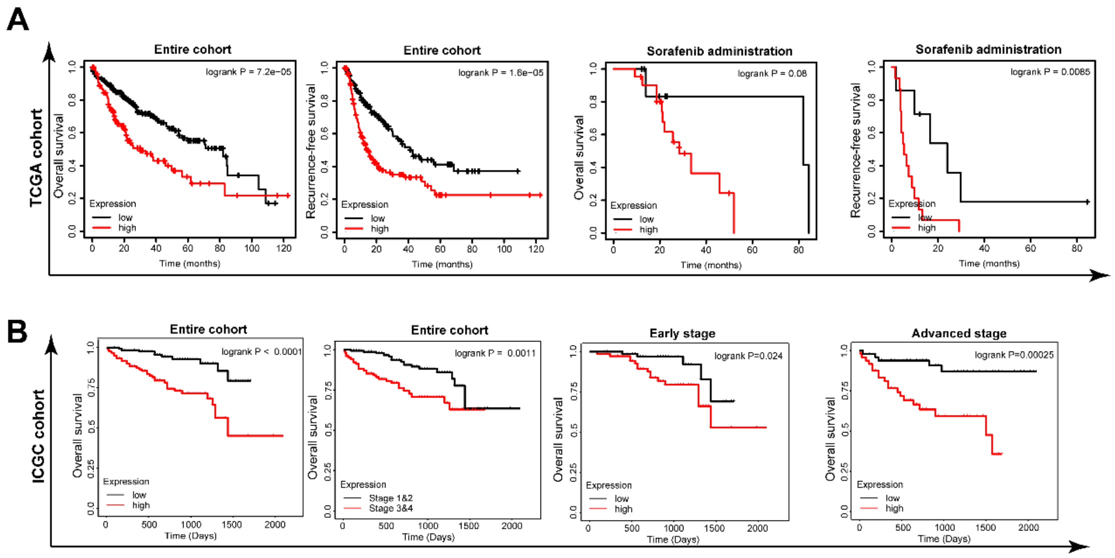

Figure S4. UBE2C indicated poor survival of HCC patients.

(A) The overall survival curves and recurrence free curves of HCC patients with high or low PRIM1 expression in entire cohort or sorafenib-treated sub-groups in TCGA LIHC dataset. (B) The overall survival curves of HCC patients with high or low PRIM1 expression in entire cohort or sub-groups in ICGC dataset.
