# Supplementary material for: DNA primase subunit 1 deteriorated progression of hepatocellular carcinoma by activating AKT/mTOR signaling and UBE2C-mediated P53 ubiquitination
Source: Cell Biosci. 2021 Feb 23;11:42. doi: 10.1186/s13578-021-00555-y (PMC7903777; doi:10.1186/s13578-021-00555-y)
Supplement: Supplementary file 5 — Additional file 5: Figure S5. UBE2C enhanced tumor growth and sorafenib resistance in HepG2-derived spheroids model. (A) The growth of the 3D spheroids derived from HepG2 in each group during Day1-5. (B) The HepG2-derived spheroids in different group treated with sorafenib. [file 13578_2021_555_MOESM5_ESM.pdf]

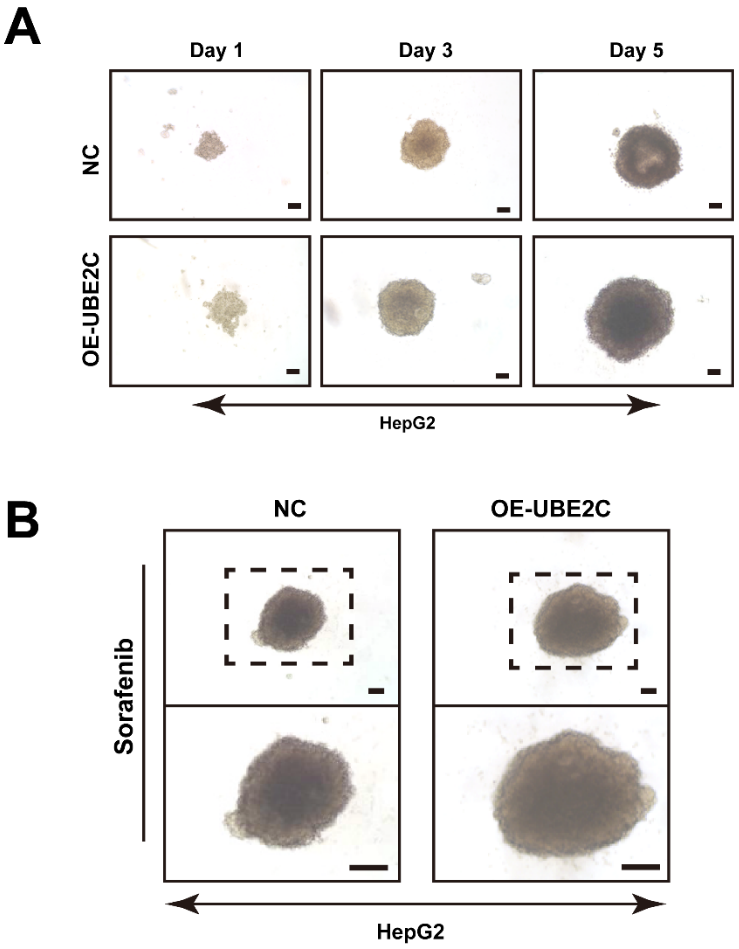

Figure S5. UBE2C enhanced tumor growth and sorafenib resistance in HepG2-derived spheroids model. (A) The growth of the 3D spheroids derived from HepG2 in each group during Day1-5. **(G)** The HepG2-derived spheroids in different group treated with sorafenib.
